# Differences in White Matter Microstructure Among Children With Developmental Coordination Disorder

**DOI:** 10.1001/jamanetworkopen.2020.1184

**Published:** 2020-03-18

**Authors:** Meisan Brown-Lum, Sara Izadi-Najafabadi, Tim F. Oberlander, Alexander Rauscher, Jill G. Zwicker

**Affiliations:** 1 Graduate Program in Rehabilitation Sciences, University of British Columbia, Vancouver, British Columbia, Canada; 2 BC Children’s Hospital Research Institute, Vancouver, British Columbia, Canada; 3 Department of Pediatrics, University of British Columbia, Vancouver, British Columbia, Canada; 4 Sunny Hill Health Centre for Children, Vancouver, British Columbia, Canada; 5 Occupational Science and Occupational Therapy, University of British Columbia, Vancouver, British Columbia, Canada

## Abstract

**Importance:**

Developmental coordination disorder (DCD) is a motor impairment that significantly interferes with activities of daily living. Little is known about the cause of DCD and how it develops, making it difficult to understand why children with DCD struggle in learning motor skills and to determine the best intervention to optimize function.

**Objective:**

To characterize white matter differences using diffusion tensor imaging in children with and without DCD.

**Design, Setting, and Participants:**

This cross-sectional study collected diffusion tensor imaging data at BC Children’s Hospital Research Institute in Vancouver, British Columbia, Canada, from September 2014 to January 2017. Using a sample of convenience, children with DCD and children without DCD aged 8 to 12 years underwent magnetic resonance imaging. Data analysis was conducted from January 2017 to January 2020.

**Main Outcomes and Measures:**

The main outcome measures were diffusion parameters, including fractional anisotropy and mean, axial, and radial diffusivity, which are thought to provide an indirect measure of white matter microstructure. Tract-based spatial statistics, a voxelwise statistical analysis of diffusion parameters, were conducted using a 2-group comparison design matrix with age and attention as covariates.

**Results:**

Thirty children without DCD (mean [SD] age, 9.9 [1.4] years; 21 [70%] boys) and 31 children with DCD (mean [SD] age, 10.1 [1.2] years; 26 [84%] boys) were included in the study. Compared with children without DCD, children with DCD were characterized by significantly lower fractional anisotropy and axial diffusivity in regions of white matter pathways associated with motor and sensorimotor processing, including the corticospinal tract (fractional anisotropy: mean [SD], 0.54 [0.03] vs 0.51 [0.03]; *P* < .001; axial diffusivity: mean [SD], 0.13 [0.98] vs 0.12 [0.46]; *P* = .01), posterior thalamic radiation at the retrolenticular part of the internal capsule (axial diffusivity: mean [SD], 0.14 [0.57] vs 0.14 [0.44]; *P* = .01), and cerebellar pathways (eg, superior cerebellar peduncle, fractional anisotropy: mean [SD], 0.49 [0.05] vs 0.46 [0.03]; *P* = .03; axial diffusivity: mean [SD], 0.14 [0.66] vs 0.14 [0.63]; *P* = .009). There were no significant differences in mean diffusivity and radial diffusivity between children with and without DCD.

**Conclusions and Relevance:**

These findings suggest that children with DCD show significant brain differences in motor and sensorimotor white matter pathways compared with children without DCD. The pattern of diffusion parameters in children with DCD suggests that axonal development may be disrupted in this neurodevelopmental disorder.

## Key Points

**Question:**

What white matter microstructural differences are associated with children with developmental coordination disorder (DCD) compared with children without DCD?

**Findings:**

In this cross-sectional study of 31 children with DCD and 30 children without DCD, widespread differences in indices of white matter microstructure, including fractional anisotropy and axial diffusivity, were observed in children with DCD compared with children without DCD.

**Meaning:**

These findings suggest that DCD does not simply represent the low end of typical motor skill ability; these children display altered brain development in sensorimotor pathways.

## Teaser

This cross-sectional study uses diffusion tensor imaging to examine microstructural differences in white matter among children with vs without developmental coordination disorder.

## Introduction

Approximately 5% to 6% of children have developmental coordination disorder (DCD), a neurodevelopmental disorder.^1^ At the core of DCD is motor impairment that significantly interferes with activities of daily living as well as academic achievement. Children with DCD have difficulty learning motor skills and performing everyday motor-related activities.^2^ Without intervention, these difficulties tend to persist into adulthood^2,3,4,5,6^ and secondary physical and psychological health concerns develop, including greater risk for obesity and coronary vascular disease, difficulty with peer relationships, anxiety, depression, and low self-esteem.^3,7,8^ Little is known about the cause of DCD and how it develops, making it difficult to understand why children with DCD struggle in learning motor skills and to determine the best intervention to optimize function. One hypothesis states that DCD is associated with pathogenesis in the central nervous system.^1^ For example, children with DCD show altered white matter development of brain pathways associated with motor and sensorimotor processing^9^; however, neural substrates of DCD have yet to be fully characterized.

Diffusion tensor imaging (DTI) is a magnetic resonance imaging (MRI) technique that enables the exploration of white matter noninvasively. The diffusion properties of white matter assessed by DTI indirectly represent physical characteristics of axonal directionality and associated white matter membrane permeability.^10,11,12^ To date, several investigations have used DTI to examine white matter differences in children with and without DCD.^9,13,14^ These studies reported reductions in indices of white matter microstructure in children with DCD compared with children without DCD. However, the studies to date have been equivocal. It is possible that the discrepancies reflect the use of different DTI acquisition protocols and preprocessing methods. It is also possible that these discrepancies reflect bias in a priori hypotheses. This study addresses this issue by applying a method of analysis that investigates the whole brain for the first time, to our knowledge. Therefore, our objective was to characterize white matter microstructural differences of the whole brain in children with DCD compared with their peers without DCD using tract-based spatial statistics (TBSS), a fully automated and simple approach that allows for analysis of the whole brain without prespecification of tracts of interest.^15^ Diffusion parameters that indirectly reflect white matter microstructure include mean diffusivity (ie, mean motion of water molecules independent of directionality), fractional anisotropy (ie, directionality of water diffusivity), axial diffusivity (ie, water diffusivity parallel to the tract), and radial diffusivity (ie, water diffusivity perpendicular to the tract).^16^

Our primary aim was to identify widespread differences across the 4 diffusion parameters that would differentiate children with and without DCD. Our secondary aim was to determine whether there was a positive association of these 4 diffusion parameters with motor performance.

## Methods

### Research Design

The Children’s and Women’s Health Centre/University of British Columbia Clinical Research Ethics Board approved this cross-sectional study. The authors were blinded to patient groupings for the data processing and initial stages of data analysis. Children were enrolled in the study following written parental informed consent and child assent. This study is reported following the Strengthening the Reporting of Observational Studies in Epidemiology (STROBE) reporting guideline.

### Study Setting

Between September 2014 and January 2017, study participants with DCD were recruited from the Developmental Coordination Disorder Research Clinic at Sunny Hill Health Centre for Children in Vancouver, British Columbia, Canada; caseloads of occupational or physical therapists in the Greater Vancouver area; and the community. Families of children with DCD as well as children without DCD were recruited through advertisements in local schools and in the community or by word of mouth. The research was conducted at the BC Children’s Hospital Research Institute. The study visit included a motor skills assessment using the Movement Assessment Battery for Children (MABC-2).^17^ Children were then screened for MRI safety followed by a session in an MRI simulator before completing a 1-hour MRI scan.

### Participants

Thirty-one children aged 8 to 12 years who met diagnostic criteria for DCD using the *Diagnostic and Statistical Manual of Mental Disorders* (Fifth Edition) were recruited for this study.^1^ Children with DCD were included if they met the following criteria: (1) a score in the 16th percentile or lower on the MABC-2^17^; (2) a score in the DCD or suspected DCD range (≤55 for children aged 8-9 years; ≤57 for children aged 10-15 years) on the Developmental Coordination Disorder Questionnaire^18^; (3) parent-reported motor difficulties from a young age; and (4) no other medical condition per parental report, clinical reports, or medical examination that could explain motor difficulties. Given that children with DCD tend to have a high co-occurrence of ADHD and more attentional issues compared with children without DCD,^19,20^ the Conners 3 Attention-Deficit/Hyperactivity Disorder (ADHD) Index^21^ was administered to quantify ADHD symptoms; a *t* score of 70 or greater is considered clinically significant. Thirty children with no history of motor difficulties who scored in the reference range on the MABC-2^17^ (ie, >25th percentile), Developmental Coordination Disorder Questionnaire,^18^ and Conners 3 ADHD Index^21^ were recruited as the cohort of children without DCD. Children were excluded if they had a medical condition that could explain their motor difficulties, such as cerebral palsy, significant intellectual disability, or visual impairment, or if they were born very preterm (ie, gestational age, <32 weeks).

### Sample Size

Using our DTI pilot data in this population,^9^ we calculated that a sample size of 27 children per group would have a power of 90% to detect a 3% difference in axial diffusivity (effect size = 1.1) with a type I error of .01.^22^ This measure of water diffusion along the length of axons in motor and sensory pathways was significantly associated with degree of motor impairment.^9^

### Neuroimaging Protocol and Analysis

All imaging was performed on a 3-T Discovery-MR 750 scanner (GE), software version DV205_R02, with a 32-channel head coil. Diffusion-weighted data were acquired using a repetition time of 7000 milliseconds, echo time of 64 milliseconds, field of view of 230 mm, matrix of 128 × 128, slice thickness of 2 mm, 32 directions, and *b* value of 1000 seconds/mm^2^. Three *b* = 0 seconds/mm^2^ volumes were acquired at the beginning of the scan. Preprocessing of diffusion data was subsequently completed using FSL brain imaging software^23^ version 5.0.9 (FMRIB Software Library). Diffusion-weighted images were corrected for motion and eddy currents, and motion-contaminated volumes were removed from the scan. Random reanalysis of 30% of the scans was completed to ensure intrarater reliability and consistency of removal of motion-contaminated volumes (intraclass correlation coefficient, 0.975; *P* < .001). All images were registered to the reference *b* = 0 seconds/mm^2^ volume to minimize image artifacts due to eddy current distortions. Lastly, nonbrain tissue was removed and the corresponding brain mask was applied to the fractional anisotropy maps.^24^ After preprocessing, reconstruction of diffusion tensors was executed.

Tract-based spatial statistics^15^ in FSL were applied to characterize whole-brain voxelwise differences in fractional anisotropy between the groups. Fractional anisotropy maps from participants were aligned with the fractional anisotropy map of the most representative participant and then affine-transformed to MNI 152 space.^15^ This step reduces the individual differences across participants. The aligned images were then used to create a mean fractional anisotropy skeleton. The fractional anisotropy skeleton threshold was set at 0.35, the maximum recommended setting to exclude peripheral tracts with high interparticipant variability or partial volume effects with gray matter.^25^ Each child’s aligned fractional anisotropy data were then projected on this skeleton, and the resulting data for each between-group analysis were entered into voxelwise cross-participant statistics (*P* < .05) using *randomize,* a permutation program used for inference (thresholding) on statistic maps when the null distribution is not known, which is effective in controlling against false-positive results.^25,26,27^ Using this skeleton-based approach in TBSS reduces the number of voxelwise tests; therefore, it poses a less severe multiple comparison problem. Moreover, we used a permutation-based approach for data analysis, which can strongly control familywise error.^15^

Independent *t* tests were run using *randomize* to examine between-group differences. A general linear model was created to compare group differences in the diffusion parameters (ie, mean diffusivity, fractional anisotropy, axial diffusivity, and radial diffusivity). Age and attention were included in the general linear model as covariates. All analyses were corrected for multiple comparisons and familywise error. Threshold-free cluster enhancement was used to help find clusters in our data without having to define clusters in a binary way^28^ and was run using 10 000 permutations. Threshold-free cluster enhancement enabled us to retain information about spatial details of extended signals (ie, each voxel’s value represents the spatial supports received from the extended signal in a clusterlike area, which makes threshold-free cluster enhancement a more sensitive and interpretable thresholding approach than cluster or voxel-based thresholding).^2^ Permutation Analysis for Linear Models^27^ was used to run associations between whole-brain fractional anisotropy values and MABC-2 percentile rank for each group separately. The anatomic location was labeled using the Johns Hopkins University ICBM-81 White-Matter Labels and White Matter Tractography Atlas.^29,30^

The original nonlinear registration from the fractional anisotropy maps was then applied to investigate mean diffusivity, axial diffusivity, and radial diffusivity maps. Each participant’s aligned mean diffusivity, axial diffusivity, and radial diffusivity data were projected on the mean fractional anisotropy skeleton.

### Statistical Analysis

Using SPSS Statistics version 24 (IBM Corp), independent *t* tests were performed to examine group differences in clinical measures, head motion, and diffusion metrics. We conducted χ^2^ tests to examine between-group differences in age and sex. Significance was set at a 1-tailed *P* < .05 and corrected for attention using the Conners *t* score. Data were analyzed for this article from January 2017 to January 2020.

## Results

### Cohort Characteristics

Of 71 enrolled children, 30 children without DCD (42%; mean [SD] age, 9.9 [1.4] years; 21 [70%] boys) and 31 children with DCD (58%; mean [SD] age, 10.1 [1.2] years; 26 [84%] boys) completed the study. Three children without DCD and 7 children with DCD did not complete the study for the following reasons: 2 children (1 without DCD; 1 with DCD) did not meet inclusion criteria, 6 children (1 without DCD; 5 with DCD) chose not to complete the scans, and 2 children (1 without DCD; 1 with DCD) were excluded owing to poor image quality. Our sample was predominantly male, which is consistent with the higher prevalence of DCD in boys.^1^ Our final sample did not differ in terms of age or sex compared with children who were excluded from analysis. There was no statistically significant difference in head motion between children without DCD and children with DCD, but children without DCD, compared with children with DCD, had significantly higher scores on the MABC-2 (median [interquartile range] percentile, 63 [31.8] vs 3.5 [8.0]; *P* = .001) and lower scores on the Conners 3 (median [interquartile range] *t* score, 45 [17.8] vs 90 [5.5]; *P* = .001) (Table 1).

**Table 1.** Cohort Characteristics

Characteristic	No DCD (n = 30)	DCD (n = 31)	*P* value
Boys, No. (%)	21 (70)	26 (84)	.20
Age, mean (SD), y	9.9 (1.4)	10.1 (1.2)	.24
MABC-2 percentile, median (IQR)	63 (31.8)	3.5 (8.0)	.001
Conners 3 ADHD Index *t* score, median (IQR)	45 (17.8)	90 (5.5)	.001
Head Motion Index score, mean (SD)	1.5 (0.34)	1.6 (0.31)	.95

Abbreviations: ADHD, attention-deficit/hyperactivity disorder; DCD, developmental coordination disorder; IQR, interquartile range; MABC-2, Movement Assessment Battery for Children, second edition.

### Group Differences in DTI Parameters Fractional Anisotropy and Axial Diffusivity

Compared with children without DCD, children with DCD had significantly lower mean fractional anisotropy that was widespread across many white matter regions. Lower fractional anisotropy was observed in white matter regions associated with the corticospinal tract (CST) (mean [SD], 0.54 [0.03] vs 0.51 [0.03]; *P* < .001), the cerebral peduncle (mean [SD], 0.56 [0.03] vs 0.54 [0.03]; *P* = .01), the cerebellar pathways (eg, superior cerebellar peduncle: mean [SD], 0.49 [0.05] vs 0.46 [0.03]; *P* = .03), the external capsule (mean [SD], 0.49 [0.03] vs 0.46 [0.02]; *P* < .001), the superior longitudinal fasciculus (mean [SD], 0.48 [0.04] vs 0.44 [0.04]; *P* < .001), and the splenium of the corpus callosum (mean [SD], 0.76 [0.03] vs 0.73 [0.03]; *P* = .002) (Table 2 and Figure). There were no regions in which the DCD group had significantly higher fractional anisotropy compared with children without DCD.

**Table 2.** Between-Group Differences in Fractional Anisotropy Values

White matter region	Side	Voxels, No.	Axis			Fractional anisotropy, mean (SD)		*P* value
			X	Y	Z	No DCD	DCD	
Corticospinal tract	R	1200	8	−22	−39	0.54 (0.03)	0.51 (0.03)	<.001
Cerebral peduncle	R	456	18	−39	−31	0.56 (0.03)	0.54 (0.03)	.01
Posterior thalamic radiation at the retrolenticular part of internal capsule	L	180	−29	−32	10	0.57 (0.03)	0.55 (0.04)	.18
Superior cerebellar peduncle	L	167	−6	−40	−27	0.49 (0.05)	0.46 (0.03)	.03
External capsule	R	628	31	13	−2	0.49 (0.03)	0.46 (0.02)	<.001
Superior longitudinal fasciculus	L	189	−30	−19	42	0.48 (0.04)	0.44 (0.04)	<.001
Splenium of the corpus callosum	L	416	−11	−36	21	0.76 (0.03)	0.73 (0.03)	.002

Abbreviations: DCD, developmental coordination disorder; L, left; R, right.

**Figure.**
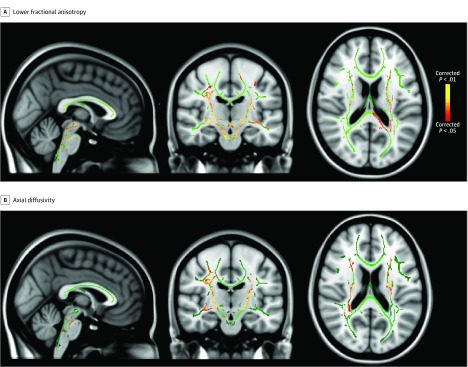
Tract-Based Spatial Statistics Analysis of Fractional Anisotropy and Axial Diffusivity A and B, Lower fractional anisotropy (A) and lower axial diffusivity (B) in children with developmental coordination disorder compared with children without development coordination disorder are shown in yellow and red, representing statistical significance. Images are displayed on the MNI 152-T1 template with the tract-based spatial statistics analysis overlaid on top of the group mean skeleton in green. Images are displayed in radiological convention in which right is left on the image.

Potential differences in mean diffusivity, axial diffusivity, and radial diffusivity among children with DCD compared with children without DCD were also explored. No statistically significant differences were found in mean diffusivity and radial diffusivity. We found statistically significant differences in mean (SD) axial diffusivity in white matter regions that overlapped with our fractional anisotropy findings (CST: 0.13 [0.98] vs 0.12 [0.46]; *P* = .01; cerebral peduncle: 0.14 [0.74] vs 0.13 [0.40]; *P* = .004; posterior thalamic radiation [PTR] at the retrolenticular part of internal capsule: 0.14 [0.57] vs 0.14 [0.44]; *P* = .01; superior cerebellar peduncle: mean [SD], 0.14 [0.66] vs 0.14 [0.63]; *P* = .009; external capsule: 0.13 [0.65] vs 0.13 [0.51]; *P* = .002) (Table 3; Figure). There were no regions where the DCD group had significantly higher axial diffusivity values compared with children without DCD. The FSL cluster command outputs were extracted for both fractional anisotropy and axial diffusivity metrics to help identify the anatomic location of the mean peak fractional anisotropy and axial diffusivity values. Mean peak clusters of fractional anisotropy and axial diffusivity greater than 100 voxels were reported (Table 2 and Table 3). After correcting for multiple comparison using familywise error, we were not able to find any significant association between MABC-2 score and fractional anisotropy values in children without DCD or children with DCD.

**Table 3.** Between-Group Differences in Axial Diffusivity Values

White matter region	Side	Voxels, No.	Axis			Axial diffusivity, mean (SD)		*P* value
			X	Y	Z	No DCD	DCD	
Corticospinal tract	R	325	9	−24	−28	0.13 (0.98)	0.12 (0.46)	.01
Cerebral peduncle	R	682	12	−23	−11	0.14 (0.74)	0.13 (0.40)	.004
Posterior thalamic radiation at the retrolenticular part of internal capsule	L	271	−28	−30	10	0.14 (0.57)	0.14 (0.44)	.01
Superior cerebellar peduncle	L	137	−4	−36	−22	0.14 (0.66)	0.14 (0.63)	.009
External capsule	R	470	34	−12	−7	0.13 (0.65)	0.13 (0.51)	.002

Abbreviations: DCD, developmental coordination disorder; L, left; R, right.

## Discussion

This cross-sectional study investigated diffusion parameters of white matter in children with and without DCD. To our knowledge, this is the first study to apply whole-brain TBSS analysis in children with DCD. Our findings suggest that diffusion parameters of white matter, including the CST, PTR, and the cerebellar pathways, are significantly different in children with DCD compared with children without DCD. Two diffusion metrics, fractional anisotropy and axial diffusivity, were found to be significantly lower in children with DCD compared with children without DCD.

### The CST

The CST is an extensive network of projection white matter pathways that connect the primary motor cortex through the corona radiata, posterior limb of the internal capsule, and cerebral peduncle to the spinal cord. The CST has a critical role in voluntary motor movement.^31^ A 2010 study by Yoshida et al^32^ found altered white matter in the CST in children with cerebral palsy, a disorder of movement and posture secondary to injury to the developing brain. Yoshida et al^32^ reported lower water diffusion along the length of CST axons was also significantly associated with gross motor outcomes. To our knowledge, the first study to investigate this motor pathway in children with DCD was in a pilot study by Zwicker et al,^9^ who reported lower axial diffusivity in the CST in children with DCD compared with children without DCD. Our study replicated this finding in a larger sample of children with DCD. However, Debrabant and colleagues^13^ did not find reduced white matter organization of the CST. This discrepancy might be a result of different analysis approaches or sample size. In this study, we used TBSS to create a study-specific skeleton for the whole brain and compare DTI parameters accordingly; however, Debrabant and colleagues^13^ did not specify how they defined their regions of interest for each participant (eg, based on a standard template or participant-specific template). Moreover, we used a voxelwise approach with permutation analysis, which effectively reduces false-positive results and increases the robustness of results^15^; Debrabant and colleagues^13^ did not specify how they calculated their DTI parameters (eg, peak fractional anisotropy, mean fractional anisotropy across multiple voxels) and used parametric analysis without permutation. Unlike Debrabant and colleagues,^13^ we used threshold-free cluster enhancement, which enabled us to retain information about spatial details of signals, increasing the sensitivity and interpretability of our results.^28^ A larger sample size in our study enabled us to detect these differences compared with the study by Debrabant et al^13^ with a smaller sample (61 participants vs 41 participants).

### The PTR

The PTR is another network of projection white matter tracts that is associated with sensory and motor processing. The PTR includes ascending and descending pathways that involve the thalamus and project to the posterior thalamic peduncle to the retrolenticular part of the internal capsule, the posterior limb of the internal capsule, and eventually projects to the premotor cortex as well as the occipital and parietal lobes.^31,33^ Altered white matter in the PTR has been reported in children with cerebral palsy^31^ and DCD.^9^ Using tractography seeds associated with the PTR in a 2012 study,^9^ we grouped the PTR, the posterior limb of the internal capsule, and the retrolenticular part of the internal capsule from our findings to be representative of the PTR. Our observations of decreased fractional anisotropy and axial diffusivity in these regions among children with DCD are consistent with previous reports.^9,13^

### The Cerebellar Pathway

The cerebellum and cerebellar peduncle contain white matter.^34^ The tracts that run through the cerebellar peduncles project to and from the spinal cord, pons and cerebral cortex, and cerebellum. These pathways assist in refining motor movements, learning new motor skills, and converting proprioceptive information into balance and posture.^35^ Our investigation found significantly lower fractional anisotropy and axial diffusivity in children with DCD, particularly in the superior cerebellar peduncle. The superior cerebellar peduncle contains efferent fibers that connect the cerebellum to the midbrain. Our findings of compromised white matter in the tracts that run through the superior cerebellar peduncle complement findings from functional MRI studies that showed underactivation of the cerebellum and parietal regions in children with DCD relative to children without DCD.^9,36^

### Impaired Interhemispheric and Intrahemispheric Communication in Children With DCD

Compared with children without DCD, we found that children with DCD had significantly lower fractional anisotropy in the superior longitudinal fasciculus and the splenium of the corpus callosum. The superior longitudinal fasciculus is part of the long association fiber network that connects the posterior temporal lobe to the motor association cortex in the frontal lobe.^29,30^ Our findings are consistent with those of Langevin et al,^14^ who reported lower fractional anisotropy in the left superior longitudinal fasciculus in children with DCD.

The posterior end of the corpus callosum, the splenium, is part of the commissural fiber network that connects the right and left hemispheres and is also associated with motor function.^37,38^ Given that the corpus callosum develops from the posterior end to the anterior end,^37,38^ low fractional anisotropy in the splenium suggests that impairment to intercortical connectivity happened early in life.^39^ However, our findings differ from those of Langevin et al,^14^ who reported lower fractional anisotropy in the left frontal region of the corpus callosum in children with DCD and ADHD and lower fractional anisotropy in the parietal region of the corpus callosum in children with DCD. Although our study did not have the power to divide the sample into an ADHD group, we did control for attention in our analyses, which could explain the discrepancy.

Our findings suggest that intrahemispheric and interhemispheric communication is impaired in children with DCD. Impaired intrahemispheric and interhemispheric connections have been proposed in other behavioral and neuroimaging studies of DCD. An electroencephalogram study^40^ showed that children with DCD had nonvoluntary mirror movements in their nonpracticing hand during a bimanual motor learning task, suggesting low interhemispheric coherence. Moreover, reduced ipsilateral silent period, as a measure of interhemispheric cortical inhibition of the primary motor cortex, supports the hypothesis of impaired interhemispheric connection in children with DCD.^41^ Izadi-Najafabadi and colleagues^42^ also reported that rehabilitation intervention was associated with improved corpus callosum structure, and therefore interhemispheric connection, in children with DCD.

### Possible Mechanisms for Low Fractional Anisotropy and Axial Diffusivity

When compared with baseline data, such as a population of children without DCD, lower fractional anisotropy values are thought to reflect impaired white matter microstructure.^43^ Fractional anisotropy is a summary measure of white matter microstructure that is sensitive to changes over time but not to the specific type of change. Many factors may affect the fractional anisotropy of white matter, including myelination, axon size, and density.^10,11,44^ In our study, we found that low fractional anisotropy values among children with DCD compared with children without DCD were accompanied by lower axial diffusivity, without significant differences in radial diffusivity or mean diffusivity. It is generally believed that low axial diffusivity reflects alterations in axon structure.^45,46,47^ This altered axon development could either be the intrinsic characteristics of axons or in the extraaxonal or extracellular space.^10^ Given that we did not find any differences in radial diffusivity or mean diffusivity, we can likely rule out demyelination or dysmyelination (driven by radial diffusivity changes) or membrane density (driven by mean diffusivity) as potential factors in the pathophysiological process of DCD. We suggest that the low fractional anisotropy and low axial diffusivity observed in children with DCD are potentially manifestations of impaired microstructure associated with altered axonal development.

Further evidence for this hypothesis is related to data acquired in infants who were born preterm. As DCD is much more common in children born preterm compared with children born at term,^48^ risk factors associated with preterm birth may shed some light on identifying possible reasons for altered white matter development found in children with DCD. For example, Zwicker et al^49^ investigated the association of antenatal, perinatal, and postnatal predictors of DCD on the development of the CST in infants born very preterm. They found that higher illness severity in the first day of life and greater exposure to painful procedures were associated with slower maturation of the CST.^49^ These findings suggest that exposures during a period of rapid brain development may influence axonal development of white matter pathways.

### Limitations

There are several limitations in this study. This is a cross-sectional study with a limited sample size that provides only a snapshot of the neural factors associated with DCD in children aged 8 to 12 years. Although the parameters derived from DTI infer microstructural differences in the white matter, a limitation of DTI is that it is sensitive but not specific in identifying white matter anomalies. The TBSS approach might also reduce the anatomic accuracy compared with higher-order diffusion models, such as constrained spherical deconvolution^50^; therefore, it is recommended that future research uses these approaches to better clarify white matter impairment in children with DCD. Another limitation is that some of the white matter tracts implicated in this study are observed in other neurodevelopmental disorders, such as ADHD. To address this, we excluded children with autism spectrum disorder and controlled for attention in our analyses to ascertain neural correlates specific to DCD. While we used a sample of convenience, we included children with co-occurring ADHD; given that approximately 50% of children with DCD also have ADHD, our sample of children aged 8 to 12 years is likely to be representative of a clinical population of children with DCD. Future research could examine differences in brain morphometry in children with and without DCD to determine whether differences exist at the macroscopic level and whether volume differences are associated with motor function.

## Conclusions

This cross-sectional study used DTI TBSS analysis to compare diffusion parameters that reflect white matter microstructure of the whole brain in children with DCD compared with children without DCD. Our results suggest that DCD is characterized by widespread differences in diffusion parameters of white matter in pathways associated with motor processing, namely the CST, PTRs, cerebellar pathways, and splenium of the corpus callosum. In addition, white matter differences were also in noted in the superior longitudinal fasciculus. Lower fractional anisotropy appears to be associated with lower axial diffusivity, which is suggestive of altered axonal development in DCD.
